# Supplementary material for: The Small RNA Universe of Capitella teleta
Source: Front Mol Biosci. 2022 Feb 25;9:802814. doi: 10.3389/fmolb.2022.802814 (PMC8915122; doi:10.3389/fmolb.2022.802814)
Supplement: Supplementary file 1 [file DataSheet1.ZIP › Supplement/known/CAPTEscaffold_14_1878.pdf]

Provisional ID	:	CAPTEscaffold_14_1878
Score total	:	2514448.5
Score for star read(s)	:	3.9
Score for read counts	:	2514438.3
Score for mfe	:	1.7
Score for randfold	:	1.6
Score for cons. seed	:	3
Total read count	:	4931974
Mature read count	:	4931392
Loop read count	:	0
Star read count	:	582

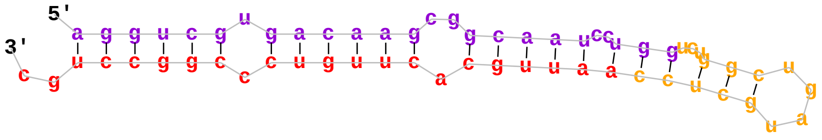

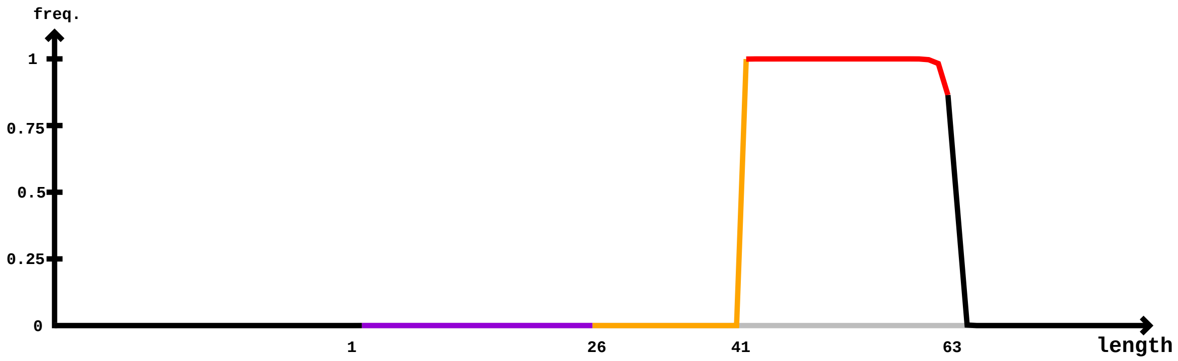

Star Mature

```
5'- gcgacucucuuauuauuacgucagugaaggaggucgugacaagcggcaauccuggucuggcugaugcuccaauugcacuugucccggccugcucagacugaccucucacguc   -3'  obs
    gcgacucucuuauuauuacgucagugaaggaggucgugacaagcggcaauccuggucuggcugaugcuccaauugcacuugucccggccugcucagacugaccucucacguc        exp
    gcgacucucuuauuauuacgucagugaaggaggucgugacaagcggcaauccuggucuggcugaugcuccaauugcacuugucccggccugcucagacugaccucucacguc        known
    ...................((((((..((.((((((.((((((..(((((..(((...(((....))))))))))).)))))).)))))).))...)))))).......... reads  mm  sample
    ..............................aggucgugacaagcggcaauccu...........................................................  13     0   seq
    ..............................aggucgugacaagcggcaauccGg..........................................................  1      1   seq
    ..............................aggucgugacaagcggcaauccug..........................................................  15     0   seq
    ..............................aggucgugacaGgcggcaauccugg.........................................................  1      1   seq
    ..............................aggucgugacaagcggcaaAccugg.........................................................  1      1   seq
    ..............................agguUgugacaagcggcaauccugg.........................................................  1      1   seq
    ..............................aggucgugacaagcggcaauccuAg.........................................................  3      1   seq
    ..............................aggucgugacaagcggcaaucUugg.........................................................  2      1   seq
    ..............................aggucgugacaagcggcaaCccugg.........................................................  1      1   seq
    ..............................Nggucgugacaagcggcaauccugg.........................................................  1      1   seq
    ..............................aggucgugacaagcggcaauccugg.........................................................  536    0   seq
    ..............................aggucgAgacaagcggcaauccugg.........................................................  1      1   seq
    ..............................aggucgugacaagcggcaauccuggA........................................................  5      1   seq
    ..............................aggucgugacaagcggcaauccuggAc.......................................................  1      1   seq
    .......................................................ucuggcugaugcuccaauugcacu.................................  36     0   seq
    .......................................................ucuggcugaugcuccaauugcacuu................................  8      0   seq
    ...................................................................uccaauugcacuugucccggccugc....................  2      0   seq
    ....................................................................ccaauugcacuugucccggccu......................  1      0   seq
    ....................................................................Ucaauugcacuugucccggccugc....................  1      1   seq
    .....................................................................caauugcacuugucccggcc.......................  3      0   seq
    .....................................................................caauugcacuugucccggccu......................  16     0   seq
    .....................................................................caauugcacuuUucccggccug.....................  1      1   seq
    .....................................................................caauugcacuugucccggccug.....................  2      0   seq
    .....................................................................caauugcacuugucccggccGg.....................  2      1   seq
    .....................................................................caauugcacuugucccCgccug.....................  1      1   seq
    .....................................................................caauugcacuugucccAgccugc....................  1      1   seq
    .....................................................................caauugcacuugucccggccAgc....................  6      1   seq
    .....................................................................caauugGacuugucccggccugc....................  1      1   seq
    .....................................................................caauugcCcuugucccggccugc....................  3      1   seq
    .....................................................................caauugcacuuUucccggccugc....................  5      1   seq
    .....................................................................caauugcacuugucccggccugc....................  264    0   seq
    .....................................................................caauugcacuugucccggccGgc....................  7      1   seq
```

Star **Mature**

```
gcgacucucuuauuauuacgucagugaaggaggucgugacaagcggcaauccuggucuggcugaugcuccaauugcacuugucccggccugcucagacugaccucucacguc
.....................................................................caauugcacuugucccggccugUu...................   1       1    seq
......................................................................aauugcacuugucccUgc........................   2       1    seq
......................................................................aauugcacuAgucccggc........................   4       1    seq
......................................................................aauAgcacuugucccggc........................   1       1    seq
......................................................................aauugcaAuugucccggc........................   1       1    seq
......................................................................aauugUacuugucccggc........................   2       1    seq
......................................................................aauugcacuugucccggA........................   18      1    seq
......................................................................Gauugcacuugucccggc........................   2       1    seq
......................................................................aauugcacuugucccggc........................   622     0    seq
......................................................................aauugcacuugucccAgcc.......................   7       1    seq
......................................................................Gauugcacuugucccggcc.......................   12      1    seq
......................................................................aauugcacuugucccggcA.......................   4       1    seq
......................................................................aauugcacuugucccgUcc.......................   1       1    seq
......................................................................aauugcacuugucccggGc.......................   2       1    seq
......................................................................aauugcacuugAcccggcc.......................   5       1    seq
......................................................................aauugcacuuguAccggcc.......................   3       1    seq
......................................................................aauugcacuugucccggcU.......................   26      1    seq
......................................................................aauCgcacuugucccggcc.......................   1       1    seq
......................................................................aauugcacuugucUcggcc.......................   9       1    seq
......................................................................aauAgcacuugucccggcc.......................   3       1    seq
......................................................................aauugcacCugucccggcc.......................   1       1    seq
......................................................................Nauugcacuugucccggcc.......................   2       1    seq
......................................................................aauugcacuuguccUggcc.......................   1       1    seq
......................................................................aauugcacuuguccAggcc.......................   4       1    seq
......................................................................aauuAcacuugucccggcc.......................   1       1    seq
......................................................................aauugcacuugucccggUc.......................   1       1    seq
......................................................................aUuugcacuugucccggcc.......................   2       1    seq
......................................................................aauugcacuugucUUggcc.......................   6       1    seq
......................................................................aauugcacuuCucccggcc.......................   1       1    seq
......................................................................aauugcGcuugucccggcc.......................   5       1    seq
......................................................................aauugcacuuguUccggcc.......................   4       1    seq
......................................................................aauugcacuuAucccggcc.......................   1       1    seq
......................................................................aGuugcacuugucccggcc.......................   2       1    seq
......................................................................aauugcaAuugucccggcc.......................   2       1    seq
......................................................................aauugUacuugucccggcc.......................   2       1    seq
......................................................................aauugcacuAgucccggcc.......................   16      1    seq
......................................................................Uauugcacuugucccggcc.......................   86      1    seq
......................................................................aauuUcacuugucccggcc.......................   1       1    seq
......................................................................aauugcacuugCcccggcc.......................   1       1    seq
......................................................................aauugcacuugucccggAc.......................   2       1    seq
......................................................................aauugcacAugucccggcc.......................   3       1    seq
......................................................................aauugcacuugucccggcG.......................   2       1    seq
......................................................................aauugcUcuugucccggcc.......................   1       1    seq
......................................................................aauugAacuugucccggcc.......................   8       1    seq
......................................................................aauugcacuugucAcggcc.......................   11      1    seq
......................................................................aauugcacuugucccgAcc.......................   1       1    seq
......................................................................aauugcacGugucccggcc.......................   2       1    seq
......................................................................aauugcacuugucccggcc.......................   13915   0    seq
......................................................................aaCugcacuugucccggcc.......................   1       1    seq
......................................................................aauugcacuCgucccggcc.......................   1       1    seq
......................................................................aauugcaGuugucccggcc.......................   1       1    seq
......................................................................aauugcacuuUucccggcc.......................   1       1    seq
......................................................................aauugcacuuguccGggcc.......................   1       1    seq
......................................................................aaAugcacuugucccggcc.......................   15      1    seq
......................................................................aauugcacuugucccgCcc.......................   2       1    seq
......................................................................aUuugcacuugucccggccu......................   4       1    seq
......................................................................aauugcacuuCucccggccu......................   3       1    seq
......................................................................aauugcacGugucccggccu......................   5       1    seq
......................................................................aaCugcacuugucccggccu......................   7       1    seq
......................................................................aauugcacuugucccgUccu......................   4       1    seq
......................................................................aauugcacuugucNcggccu......................   1       1    seq
......................................................................aauugcacuugucccggccG......................   267     1    seq
......................................................................aauugcacuuguGccggccu......................   6       1    seq
......................................................................aauugcacuugucccUgccu......................   5       1    seq
......................................................................aNuugcacuugucccggccu......................   2       1    seq
......................................................................aauugcacuugucccggccu......................   69534   0    seq
......................................................................Cauugcacuugucccggccu......................   2       1    seq
......................................................................aaGugcacuugucccggccu......................   1       1    seq
......................................................................aauugcacuuguUccggccu......................   18      1    seq
......................................................................aauugcacuGgucccggccu......................   8       1    seq
```

```
                              Star                                      Mature
gcgacucucuuauuauuacgucagugaaggaggucgugacaagcggcaauccuggucuggcugaugcuccaauugcacuugucccggccugcucagacugaccucucacguc
......................................................................aauugcacuCgucccggccu......................  7       1   seq
......................................................................aauugcacuugucccAgccu......................  24      1   seq
......................................................................Gauugcacuugucccggccu......................  50      1   seq
......................................................................aauugcacAugucccggccu......................  17      1   seq
......................................................................aauugcacuugucGcggccu......................  4       1   seq
......................................................................aauGgcacuugucccggccu......................  1       1   seq
......................................................................aauugcacuugucccggAcu......................  12      1   seq
......................................................................aauugcacuugucccggcGu......................  3       1   seq
......................................................................aauugcacuugucccggccC......................  12      1   seq
......................................................................aauugcacCugucccggccu......................  3       1   seq
......................................................................aauugcacuuUucccggccu......................  14      1   seq
......................................................................aauugAacuugucccggccu......................  23      1   seq
......................................................................aauAgcacuugucccggccu......................  29      1   seq
......................................................................Uauugcacuugucccggccu......................  3       1   seq
......................................................................aauugcGcuugucccggccu......................  21      1   seq
......................................................................aCuugcacuugucccggccu......................  1       1   seq
......................................................................aauugcUcuugucccggccu......................  6       1   seq
......................................................................aauugcacuAgucccggccu......................  88      1   seq
......................................................................aauugUacuugucccggccu......................  10      1   seq
......................................................................aauugcaGuugucccggccu......................  3       1   seq
......................................................................aauugcacuugucAcggccu......................  32      1   seq
......................................................................aauugcacuugucccggcAu......................  7       1   seq
......................................................................aauCgcacuugucccggccu......................  12      1   seq
......................................................................aauuAcacuugucccggccu......................  8       1   seq
......................................................................aauugcacuugucccCgccu......................  3       1   seq
......................................................................aauugcacuugCcccggccu......................  8       1   seq
......................................................................aauugGacuugucccggccu......................  5       1   seq
......................................................................aauugcacuuguccUggccu......................  57      1   seq
......................................................................aGuugcacuugucccggccu......................  2       1   seq
......................................................................aauugcacuugucccggccA......................  165     1   seq
......................................................................Nauugcacuugucccggccu......................  26      1   seq
......................................................................aauugcacuugucUcggccu......................  124     1   seq
......................................................................aauugcacuugucccggcUu......................  13      1   seq
......................................................................aauugcacuugucccggGcu......................  2       1   seq
......................................................................aauugcaAuugucccggccu......................  7       1   seq
......................................................................aaAugcacuugucccggccu......................  80      1   seq
......................................................................aauugcaUuugucccggccu......................  4       1   seq
......................................................................aauuCcacuugucccggccu......................  3       1   seq
......................................................................aauugcacuuAucccggccu......................  11      1   seq
......................................................................aauugcacuugGcccggccu......................  5       1   seq
......................................................................aauugcacuuguAccggccu......................  28      1   seq
......................................................................aauugcacuugucccgAccu......................  10      1   seq
......................................................................aauugcacuuguccGggccu......................  9       1   seq
......................................................................aauugcacuugucccggUcu......................  8       1   seq
......................................................................aauuUcacuugucccggccu......................  4       1   seq
......................................................................aauugcCcuugucccggccu......................  3       1   seq
......................................................................aauugcacuuguccAggccu......................  20      1   seq
......................................................................aauugcacuugAcccggccu......................  35      1   seq
......................................................................aauugcacuugucccgCccu......................  4       1   seq
......................................................................aauugcacuAgucccggccug.....................  271     1   seq
......................................................................Cauugcacuugucccggccug.....................  8       1   seq
......................................................................aauugUacuugucccggccug.....................  88      1   seq
......................................................................aauugcacuugucccAgccug.....................  207     1   seq
......................................................................aauugcacuugucccUgccug.....................  32      1   seq
......................................................................aauugcacuuguGccggccug.....................  35      1   seq
......................................................................aauugcacuuguAccggccug.....................  182     1   seq
......................................................................aauugcacuugucccggccuN.....................  3       1   seq
......................................................................aCuugcacuugucccggccug.....................  1       1   seq
......................................................................aauugcacuuguccAggccug.....................  222     1   seq
......................................................................Gauugcacuugucccggccug.....................  724     1   seq
......................................................................aaGugcacuugucccggccug.....................  15      1   seq
......................................................................aauugcacuugucccggccug.....................  571031  0   seq
......................................................................aauugcacuugucGcggccug.....................  50      1   seq
......................................................................aauugcacAugucccggccug.....................  96      1   seq
......................................................................Nauugcacuugucccggccug.....................  139     1   seq
......................................................................aauugcacuuguccUggccug.....................  354     1   seq
......................................................................aauugcCcuugucccggccug.....................  31      1   seq
......................................................................aauAgcacuugucccggccug.....................  188     1   seq
......................................................................aauugcacuugucccggGcug.....................  51      1   seq
......................................................................aauugcacuugucccggcAug.....................  87      1   seq
```

Star **Mature**

```
gcgacucucuuauuauuacgucagugaaggaggucgugacaagcggcaauccuggucuggcugaugcuccaauugcacuugucccggccugcucagacugaccucucacguc
......................................................................aauugcacuugucUcggccug.....................     464       1    seq
......................................................................aauugcaUuugucccggccug.....................     20        1    seq
......................................................................aauGgcacuugucccggccug.....................     7         1    seq
......................................................................aauuUcacuugucccggccug.....................     47        1    seq
......................................................................aauugcacuugucccggccCg.....................     82        1    seq
......................................................................aaNugcacuugucccggccug.....................     1         1    seq
......................................................................aauugcacGugucccggccug.....................     55        1    seq
......................................................................aauugcacuugucccgNccug.....................     1         1    seq
......................................................................aNuugcacuugucccggccug.....................     11        1    seq
......................................................................aUuugcacuugucccggccug.....................     43        1    seq
......................................................................aauugcacuugucccggccAg.....................     108       1    seq
......................................................................aauugcacuuUucccggccug.....................     34        1    seq
......................................................................aauugGacuugucccggccug.....................     37        1    seq
......................................................................aauugcaGuugucccggccug.....................     16        1    seq
......................................................................aauugcNcuugucccggccug.....................     3         1    seq
......................................................................aauugcacuugucccCgccug.....................     24        1    seq
......................................................................aauugcacuugucccggUcug.....................     71        1    seq
......................................................................aauugcacuugucccgAccug.....................     64        1    seq
......................................................................aauugcacuuguccGggccug.....................     71        1    seq
......................................................................aauugcacuugucccggccuC.....................     152       1    seq
......................................................................aaCugcacuugucccggccug.....................     28        1    seq
......................................................................aauugcacCugucccggccug.....................     126       1    seq
......................................................................aauugcacuugucccggAcug.....................     109       1    seq
......................................................................aauugcacuugucccggcUug.....................     121       1    seq
......................................................................aauuAcacuugucccggccug.....................     67        1    seq
......................................................................aauugcacuugAcccggccug.....................     226       1    seq
......................................................................aauugcacuugucccggccuA.....................     91        1    seq
......................................................................aauCgcacuugucccggccug.....................     73        1    seq
......................................................................aauugAacuugucccggccug.....................     317       1    seq
......................................................................aauugcacuuCucccggccug.....................     26        1    seq
......................................................................aauugcacuuguUccggccug.....................     66        1    seq
......................................................................aauugcacuugucccgCccug.....................     31        1    seq
......................................................................aauuCcacuugucccggccug.....................     24        1    seq
......................................................................aauugcUcuugucccggccug.....................     51        1    seq
......................................................................aauugcacuugucccggcGug.....................     40        1    seq
......................................................................aauugcacuugGcccggccug.....................     26        1    seq
......................................................................aauugcacuugucccgUccug.....................     32        1    seq
......................................................................aauugcacuuNucccggccug.....................     2         1    seq
......................................................................aauugcacuGgucccggccug.....................     13        1    seq
......................................................................aauugcacuugucAcggccug.....................     436       1    seq
......................................................................aGuugcacuugucccggccug.....................     53        1    seq
......................................................................aauugcacuugucccNgccug.....................     1         1    seq
......................................................................aauugcaAuugucccggccug.....................     77        1    seq
......................................................................Uauugcacuugucccggccug.....................     51        1    seq
......................................................................aaAugcacuugucccggccug.....................     560       1    seq
......................................................................aauuNcacuugucccggccug.....................     2         1    seq
......................................................................aauugcacuugucccggccGg.....................     4124      1    seq
......................................................................aauugcacuCgucccggccug.....................     67        1    seq
......................................................................aauugcacuNgucccggccug.....................     4         1    seq
......................................................................aauugcGcuugucccggccug.....................     122       1    seq
......................................................................aauugcacuugCcccggccug.....................     161       1    seq
......................................................................aauugcacuuAucccggccug.....................     98        1    seq
......................................................................aauugcacuugucccggccuU.....................     252       1    seq
......................................................................aauugNacuugucccggccug.....................     27        1    seq
......................................................................aauugcacuugucccUgccugc....................     231       1    seq
......................................................................aauugcacuugucccgNccugc....................     6         1    seq
......................................................................aauugcacuGgucccggccugc....................     279       1    seq
......................................................................aauugcacNugucccggccugc....................     10        1    seq
......................................................................aauugcacuugucccgUccugc....................     315       1    seq
......................................................................aauugcacuugucNcggccugc....................     10        1    seq
......................................................................aauugcacCugucccggccugc....................     2085      1    seq
......................................................................aauGgcacuugucccggccugc....................     108       1    seq
......................................................................aauugcacuuguccAggccugc....................     1374      1    seq
......................................................................aauugcacuugucccggccuUc....................     274       1    seq
......................................................................aauugcacuugucAcggccugc....................     2790      1    seq
......................................................................Gauugcacuugucccggccugc....................     5091      1    seq
......................................................................aauugUacuugucccggccugc....................     484       1    seq
......................................................................aauugcacuAgucccggccugc....................     2123      1    seq
......................................................................aauugcacuugucccggccugc....................     4031036   0    seq
......................................................................aauugcGcuugucccggccugc....................     1242      1    seq
```

```
                              Star                                       Mature
gcgacucucuuauuauuacgucagugaaggaggucgugacaagcggcaauccuggucuggcugaugcuccaauugcacuugucccggccugcucagacugaccucucacguc
......................................................................aauugcacuugucUcggccugc....................  3309   1  seq
......................................................................aauugcacuugGcccggccugc....................  212    1  seq
......................................................................Uauugcacuugucccggccugc....................  403    1  seq
......................................................................aauCgcacuugucccggccugc....................  731    1  seq
......................................................................aauugcacuugucccggccugN....................  11     1  seq
......................................................................aauugcacuuguccNggccugc....................  1      1  seq
......................................................................Cauugcacuugucccggccugc....................  78     1  seq
......................................................................aauugcacuugucccggccCgc....................  540    1  seq
......................................................................aauugcaNuugucccggccugc....................  3      1  seq
......................................................................aauugcacuuguAccggccugc....................  1393   1  seq
......................................................................aauugcCcuugucccggccugc....................  252    1  seq
......................................................................aauugcacuuguccUggccugc....................  2520   1  seq
......................................................................aauugcacuuguGccggccugc....................  265    1  seq
......................................................................aauugcacuugucccggcUugc....................  742    1  seq
......................................................................aauuCcacuugucccggccugc....................  224    1  seq
......................................................................aauugcacuugucccggccNgc....................  6      1  seq
......................................................................aauugcaUuugucccggccugc....................  272    1  seq
......................................................................aauugcacAugucccggccugc....................  718    1  seq
......................................................................aauugcaGuugucccggccugc....................  168    1  seq
......................................................................aauugcacuNgucccggccugc....................  33     1  seq
......................................................................aauugcacuugucccAgccugc....................  1663   1  seq
......................................................................aauugcacuugucccgAccugc....................  629    1  seq
......................................................................aCuugcacuugucccggccugc....................  12     1  seq
......................................................................aauugcacuugucccggccugG....................  1620   1  seq
......................................................................aauugcacuugucccggccGgc....................  213    1  seq
......................................................................aauugcacuugucccgCccugc....................  214    1  seq
......................................................................aauugcacuugucccggccuAc....................  504    1  seq
......................................................................aauugcacuugucccggccugU....................  47228  1  seq
......................................................................aGuugcacuugucccggccugc....................  379    1  seq
......................................................................aauugNacuugucccggccugc....................  132    1  seq
......................................................................aauugcacuugucGcggccugc....................  334    1  seq
......................................................................aauugcacuugucccCgccugc....................  223    1  seq
......................................................................aauugcacuCgucccggccugc....................  611    1  seq
......................................................................aauugcUcuugucccggccugc....................  549    1  seq
......................................................................aaCugcacuugucccggccugc....................  399    1  seq
......................................................................aauugAacuugucccggccugc....................  2138   1  seq
......................................................................aauugcacuuCucccggccugc....................  239    1  seq
......................................................................aauugcacGugucccggccugc....................  296    1  seq
......................................................................aauugcacuugucccggccuCc....................  252    1  seq
......................................................................aauugcacuugucccggccuNc....................  34     1  seq
......................................................................aaNugcacuugucccggccugc....................  15     1  seq
......................................................................aauAgcacuugucccggccugc....................  1536   1  seq
......................................................................aauugcacuuNucccggccugc....................  7      1  seq
......................................................................aauugcacuuguUccggccugc....................  936    1  seq
......................................................................aauugcacuugucccggcAugc....................  541    1  seq
......................................................................aauugcacuugucccggcGugc....................  245    1  seq
......................................................................aauugcNcuugucccggccugc....................  2      1  seq
......................................................................aauugcacuuUucccggccugc....................  353    1  seq
......................................................................aNuugcacuugucccggccugc....................  115    1  seq
......................................................................aauugcacuugucccNgccugc....................  6      1  seq
......................................................................aauugcacuugCcccggccugc....................  694    1  seq
......................................................................aauugcaAuugucccggccugc....................  520    1  seq
......................................................................aauugGacuugucccggccugc....................  225    1  seq
......................................................................aauugcacuugucccggccAgc....................  738    1  seq
......................................................................aauuUcacuugucccggccugc....................  496    1  seq
......................................................................aauuAcacuugucccggccugc....................  564    1  seq
......................................................................aaAugcacuugucccggccugc....................  3930   1  seq
......................................................................aauugcacuuAucccggccugc....................  610    1  seq
......................................................................aauugcacuugucccggcNugc....................  6      1  seq
......................................................................aauugcacuugAcccggccugc....................  1931   1  seq
......................................................................aUuugcacuugucccggccugc....................  274    1  seq
......................................................................aauugcacuuguccGggccugc....................  384    1  seq
......................................................................aauugcacuugucccggAcugc....................  779    1  seq
......................................................................aauugcacuugucccggUcugc....................  424    1  seq
......................................................................aauugcacuugucccggGcugc....................  322    1  seq
......................................................................aaGugcacuugucccggccugc....................  38     1  seq
......................................................................Nauugcacuugucccggccugc....................  995    1  seq
......................................................................aauugcacuugucccggccugA....................  5093   1  seq
......................................................................aauugcacuuguccUggccugcu...................  47     1  seq
......................................................................aauugcacuugucccggccugAu...................  136    1  seq
```

```
                              Star                                   Mature
gcgacucucuuauuauuacgucagugaaggaggucgugacaagcggcaauccuggucuggcugaugcuccaauugcacuugucccggccugcucagacugaccucucacguc
......................................................................aauuAcacuugucccggccugcu...................   11      1   seq
......................................................................aaGugcacuugucccggccugcu...................   1       1   seq
......................................................................aauugcacuugucccUgccugcu...................   5       1   seq
......................................................................aauugUacuugucccggccugcu...................   11      1   seq
......................................................................aauugcacuugCcccggccugcu...................   10      1   seq
......................................................................aauugcacuuguGccggccugcu...................   5       1   seq
......................................................................aauugcacuugucccggccGgcu...................   5       1   seq
......................................................................aaNugcacuugucccggccugcu...................   1       1   seq
......................................................................aauugcacuugucUcggccugcu...................   66      1   seq
......................................................................aauugcacuugucccggccuAcu...................   7       1   seq
......................................................................aauugcacuCgucccggccugcu...................   11      1   seq
......................................................................aauugcacuugucccggccuUcu...................   7       1   seq
......................................................................aGuugcacuugucccggccugcu...................   9       1   seq
......................................................................aauugcacuuguAccggccugcu...................   31      1   seq
......................................................................Gauugcacuugucccggccugcu...................   141     1   seq
......................................................................aauugcacuugGcccggccugcu...................   5       1   seq
......................................................................aauAgcacuugucccggccugcu...................   46      1   seq
......................................................................aCuugcacuugucccggccugcu...................   2       1   seq
......................................................................aauugNacuugucccggccugcu...................   4       1   seq
......................................................................aauugcacuugucGcggccugcu...................   9       1   seq
......................................................................aauugcacuugAcccggccugcu...................   37      1   seq
......................................................................aauugcGcuugucccggccugcu...................   22      1   seq
......................................................................aauugcacuuAucccggccugcu...................   13      1   seq
......................................................................aauugcacuugucccgCccugcu...................   11      1   seq
......................................................................aauugcacGugucccggccugcu...................   6       1   seq
......................................................................aauugcacuuUucccggccugcu...................   40      1   seq
......................................................................aauugcacuugucccggccugUu...................   1803    1   seq
......................................................................aauugcacuuguccGggccugcu...................   7       1   seq
......................................................................Uauugcacuugucccggccugcu...................   9       1   seq
......................................................................aauugcacuugucccCgccugcu...................   6       1   seq
......................................................................aauugcacuugucccggccugcA...................   10731   1   seq
......................................................................aauugcacuuguccAggccugcu...................   40      1   seq
......................................................................aauugcaGuugucccggccugcu...................   4       1   seq
......................................................................aauugcacuugucccggccAgcu...................   13      1   seq
......................................................................aauugcaUuugucccggccugcu...................   3       1   seq
......................................................................aauugcacuugucccggccCgcu...................   11      1   seq
......................................................................aauugcacuugucccggGcugcu...................   8       1   seq
......................................................................aauugcacuugucccggccugcG...................   329     1   seq
......................................................................aauugcacuugucccggAcugcu...................   13      1   seq
......................................................................aauugGacuugucccggccugcu...................   12      1   seq
......................................................................aauugcacuugucccggcGugcu...................   5       1   seq
......................................................................aauugcacuugucccggccugGu...................   10      1   seq
......................................................................aauugcacuugucccggcUugcu...................   15      1   seq
......................................................................aUuugcacuugucccggccugcu...................   9       1   seq
......................................................................aauugcacuugucccggccugcu...................   97209   0   seq
......................................................................aauugAacuugucccggccugcu...................   42      1   seq
......................................................................aauugcacAugucccggccugcu...................   20      1   seq
......................................................................Nauugcacuugucccggccugcu...................   25      1   seq
......................................................................Cauugcacuugucccggccugcu...................   2       1   seq
......................................................................aauugcacuAgucccggccugcu...................   44      1   seq
......................................................................aaCugcacuugucccggccugcu...................   2       1   seq
......................................................................aauuCcacuugucccggccugcu...................   4       1   seq
......................................................................aauugcacuugucccggccugcN...................   7       1   seq
......................................................................aauugcacuuguUccggccugcu...................   10      1   seq
......................................................................aauugcacuugucccggcAugcu...................   109     1   seq
......................................................................aauugcCcuugucccggccugcu...................   4       1   seq
......................................................................aauugcacCugucccggccugcu...................   5       1   seq
......................................................................aauugcacuugucccAgccugcu...................   33      1   seq
......................................................................aaAugcacuugucccggccugcu...................   82      1   seq
......................................................................aNuugcacuugucccggccugcu...................   3       1   seq
......................................................................aauGgcacuugucccggccugcu...................   1       1   seq
......................................................................aauugcUcuugucccggccugcu...................   8       1   seq
......................................................................aauugcacuugucccggccuCcu...................   5       1   seq
......................................................................aauugcaAuugucccggccugcu...................   13      1   seq
......................................................................aauuUcacuugucccggccugcu...................   7       1   seq
......................................................................aauugcacuugucccggUcugcu...................   9       1   seq
......................................................................aauugcacuugucccggccugNu...................   1       1   seq
......................................................................aauCgcacuugucccggccugcu...................   17      1   seq
......................................................................aauugcacuugucAcggccugcu...................   59      1   seq
......................................................................aauugcacuugucNcggccugcu...................   1       1   seq
```

```
                             Star                                      Mature
gcgacucucuuauuauuacgucagugaaggaggucgugacaagcggcaauccuggucuggcugauguccaauugcacuugucccggccugcucagacugaccucucacguc
.....................................................................aauugcacuugucccgUccugcu...................  9     1  seq
.....................................................................aauugcacuugucccgAccugcu...................  13    1  seq
.....................................................................aauugcacuGgucccggccugcu...................  20    1  seq
.....................................................................aauugcacuugucAcggccugcuc..................  1     1  seq
.....................................................................aauugcacuugucccggccugcuc..................  11    0  seq
.....................................................................aauugcacuugucccggccugcuU..................  9253  1  seq
.....................................................................aauugcacuugucccggccugcAc..................  14    1  seq
.....................................................................aauugcacuugucccggccugcuA..................  172   1  seq
.....................................................................aauugcacuugucccggccugcuG..................  5     1  seq
.....................................................................aauugcacuugucccggccugcucU.................  2     1  seq
.....................................................................aauugcacuugucccggccugcuAa.................  25    1  seq
.....................................................................aauugcacuugucccggccugcuUa.................  2     1  seq
.....................................................................aauugcacuugucccggccugcuca.................  9     0  seq
......................................................................auugcacuugucccggcc.......................  62    0  seq
......................................................................aAugcacuugucccggcc.......................  1     1  seq
......................................................................auugcacuugucccggccG......................  1     1  seq
......................................................................auugcacuugucccggccu......................  35    0  seq
......................................................................auugcacuugucccggccug.....................  101   0  seq
......................................................................aAugcacuugucccggccug.....................  10    1  seq
......................................................................auugcacuugucAcggccugc....................  3     1  seq
......................................................................aAugcacuugucccggccugc....................  39    1  seq
......................................................................auugcacuugucccggAcugc....................  1     1  seq
......................................................................auugcacuuguccGggccugc....................  2     1  seq
......................................................................auugcUcuugucccggccugc....................  1     1  seq
......................................................................auugcacuugucccggccugU....................  1     1  seq
......................................................................auugcacAugucccggccugc....................  2     1  seq
......................................................................auugcacuugucccggcUugc....................  1     1  seq
......................................................................auugcacuugucccggccugc....................  1606  0  seq
......................................................................auAgcacuugucccggccugc....................  2     1  seq
......................................................................Guugcacuugucccggccugc....................  25    1  seq
......................................................................aGugcacuugucccggccugc....................  1     1  seq
......................................................................auugcacuugAcccggccugc....................  2     1  seq
......................................................................auugcacuuguAccggccugc....................  1     1  seq
......................................................................auugcacuugucccgAccugc....................  1     1  seq
......................................................................auugcacuuAucccggccugc....................  1     1  seq
......................................................................aAugcacuugucccggccugcu...................  2     1  seq
......................................................................Uuugcacuugucccggccugcu...................  1     1  seq
......................................................................Guugcacuugucccggccugcu...................  1     1  seq
......................................................................auugcacuugucccggccugcu...................  181   0  seq
......................................................................auugcacuuguccAggccugcu...................  1     1  seq
......................................................................auugcacuugucccggccugAu...................  1     1  seq
......................................................................auugcacuuguccUggccugcu...................  1     1  seq
......................................................................auugcacuugucccggccugcA...................  115   1  seq
......................................................................auugcacuugucccggccugUu...................  1     1  seq
......................................................................auugcacuugucccggccugcuU..................  209   1  seq
......................................................................auugcacuugucccggccugcuG..................  1     1  seq
.......................................................................uugcacuugucccggccu......................  11    0  seq
.......................................................................uugcacuugucccggccug.....................  1     0  seq
.......................................................................uugcacuugucAcggccugc....................  1     1  seq
.......................................................................Augcacuugucccggccugc....................  1     1  seq
.......................................................................uugcacuugucccggccugU....................  1     1  seq
.......................................................................uugcacuugucccggccugc....................  298   0  seq
.......................................................................uugcacuuguccUggccugc....................  1     1  seq
.......................................................................uAgcacuugucccggccugc....................  1     1  seq
.......................................................................uugcacuAgucccggccugc....................  2     1  seq
.......................................................................uugcacuugucccggccugcu...................  1     0  seq
.......................................................................uugcaUuugucccggccugcu...................  1     1  seq
.......................................................................uugcacuugucccggccugcA...................  1     1  seq
........................................................................ugcacuugucccggccugc....................  27    0  seq
........................................................................ugcacuugucccggccugcuG..................  1     1  seq
.........................................................................gcacuugucccggccugU....................  1     1  seq
.........................................................................gcacuugucccggccugc....................  188   0  seq
.........................................................................gcacuugucccggccugcu...................  1     0  seq
```
